# Construction of BAC contig maps of homoeologous chromosomes A12 and D12 of *Gossypium hirsutum* L. acc. TM-1

**DOI:** 10.1186/s13039-015-0158-z

**Published:** 2015-07-28

**Authors:** Yanhui Lv, Dan Ma, Wenhua Liang, Yuanda Lv, Wangzhen Guo, Yan Hu, Tianzhen Zhang

**Affiliations:** State Key Laboratory of Crop Genetics & Germplasm Enhancement, Hybrid Cotton R & D Engineering Research Center, MOE, Nanjing Agricultural University, Nanjing, 210095 China

**Keywords:** Contig, BAC, Fingerprint, Homoeologous chromosome, Cotton, Genomics

## Abstract

**Background:**

The *Gossypium hirsutum* homoeologous chromosome 12 encodes important genes that contribute to fiber fuzz, lethality, gland development and male sterility. In this study a physical map of the cotton TM-1 chromosome 12 was constructed. A number of large-insert cotton genome libraries are available, and genome-wide physical mapping using large insert segments combined with bacterial cloning is a thriving area of genome research. However, sequencing of the cotton genome is difficult due to sequence repeats and homoeologous regions. In order to effectively distinguish the homologous segments, a new method for adjusting the parameters of the FPC software was applied for contig map construction.

**Results:**

All available markers on chromosomes A12 and D12 were used to screen the TM-1 BAC library by PCR. A total of 775 clones (387 for A12, 388 for D12) were obtained using *Hin*d III fingerprinting and used for construction of the contig map. Seven pairs of SSR markers located on A12 and D12 were chosen for contig analysis. Following optimization of the tolerance (10) and cutoff (1e-12) parameters, combining all clones from A12 and D12 produced two separate contigs.

**Conclusions:**

The BAC contig map of chromosomes A12 and D12 was constructed and FPC software parameters were optimized for analysis. The resulting approach is a powerful platform for genome-wide and evolutionary research on cotton.

## Background

BACs (bacterial artificial chromosomes) are important resources for map-based cloning and large-scale sequencing of complex genomes due to their ability to stably maintain large DNA fragments that facilitate easy manipulation [1, 2]. BAC clones can be arranged into contigs which are contiguous, gap-free overlapping clones [3, 4] which can be used to identify the minimum tilling path fingerprinted contigs required for further gene cloning. BAC libraries have been constructed from various different cotton species, including *Maxxa* [5], *Suyuan7235* [6], *Zhongmiansuo 12* [7], *0-613-2R* [8], and *Pima 90–53* [9]. These libraries can be used to construct a high quality physical map of the cotton genome.

Cotton is the source of the world most important plant-derived fiber, and is also an important oilseed crop, as well as a model species for the study of plant polyploidy, cellulose biosynthesis and cell wall biogenesis [10]. The genus *Gossypium* consists of over 50 species, including 40–45 diploids (2n = 2 × = 26) and five allotetraploids (2n = 4 × = 52) [11] that were formed from separate A and D genomes through polyploidization 1–2 million years ago [12–14]. The tetraploid cotton genome contains 13 pairs of homologous chromosomes [15–17] which have been incorporated into specific BAC clones, and a new chromosome nomenclature for tetraploid cotton has been proposed [18]. A12 and D12 chromosomes in allotetraploid cotton are among the most important pairs of homoeologous chromosomes, and include the alleles (*N*_*1*_,*n*_*2*_) at naked seeds [19], *Le*_*1*_, *Le*_*2*_ at hybrid lethality [20, 21], *gl*_*2*_, *gl*_*3*_ at gossypol glanding, *ne*_*1*_,*ne*_*2*_ at mecariless, *Bw*_*1*_, *Bw*_*2*_ at withering bract, *ms*_*8*_,*ms*_*9*_ at male sterile [22–26]. For map-based cloning and whole-genome sequencing of tetraploid cotton, a BAC library for *G. hirsutum* acc. TM-1 has been constructed [8, 27, 28]. Furthermore, a high-density gene-rich genetic map containing 2247 loci and covering 3540.4-cM, with an average inter-marker distance of 1.58-cM, has been constructed from the BC_1_ (TM-1 × Hai7124) cross [29–34].

In this study, to facilitate construction of BAC contig maps for the *G. hirsutum* L. Acc. TM-1 homoeologous chromosomes A12 and D12, the molecular markers identified in the aforementioned genetic map were used to screen the BAC library. BACs containing genetic markers that produced characteristic fingerprints when digested with the restriction enzyme *Hin*d III were used to build contigs using the FPC software. A BAC fingerprint-based contig map of both A12 and D12 was successfully constructed by adjusting the tolerance and cutoff parameters in FPC. This approach will facilitate future map-based cloning of important cotton genes and expand our understanding of the relationships between the genetic and physical maps of A12 and D12.

## Results

### Identifying BAC clones containing genes from chromosomes A12 and D12

BAC library screening was based on the published linkage map [33], and a total of 101 and 124 SSR markers from tetraploid cotton homoeologous chromosomes A12 and D12, respectively, were selected for screening the TM-1 library by a PCR-based method [27]. The PCR products were abundant and well-resolved, and positive BAC clones were identified by the SSR marker. In total, 775 positive BAC clones were screened out using the SSR markers, and each marker identified and average of five clones.

**Fig. 1:**
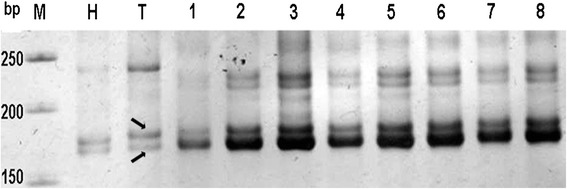
Identification of BAC library with dPL0240. The dPL0240-based PCR product of parents and positive BAC clones is denoted by the single arrow. The arrow denotes a positive BAC clone that is screened from TM-1 BAC library. The positive BAC clone has the same band as the parent(TM-1); M:Marker,H:Hai7124,T:TM-1,1-8: Positive BAC clone

**Fig. 2:**
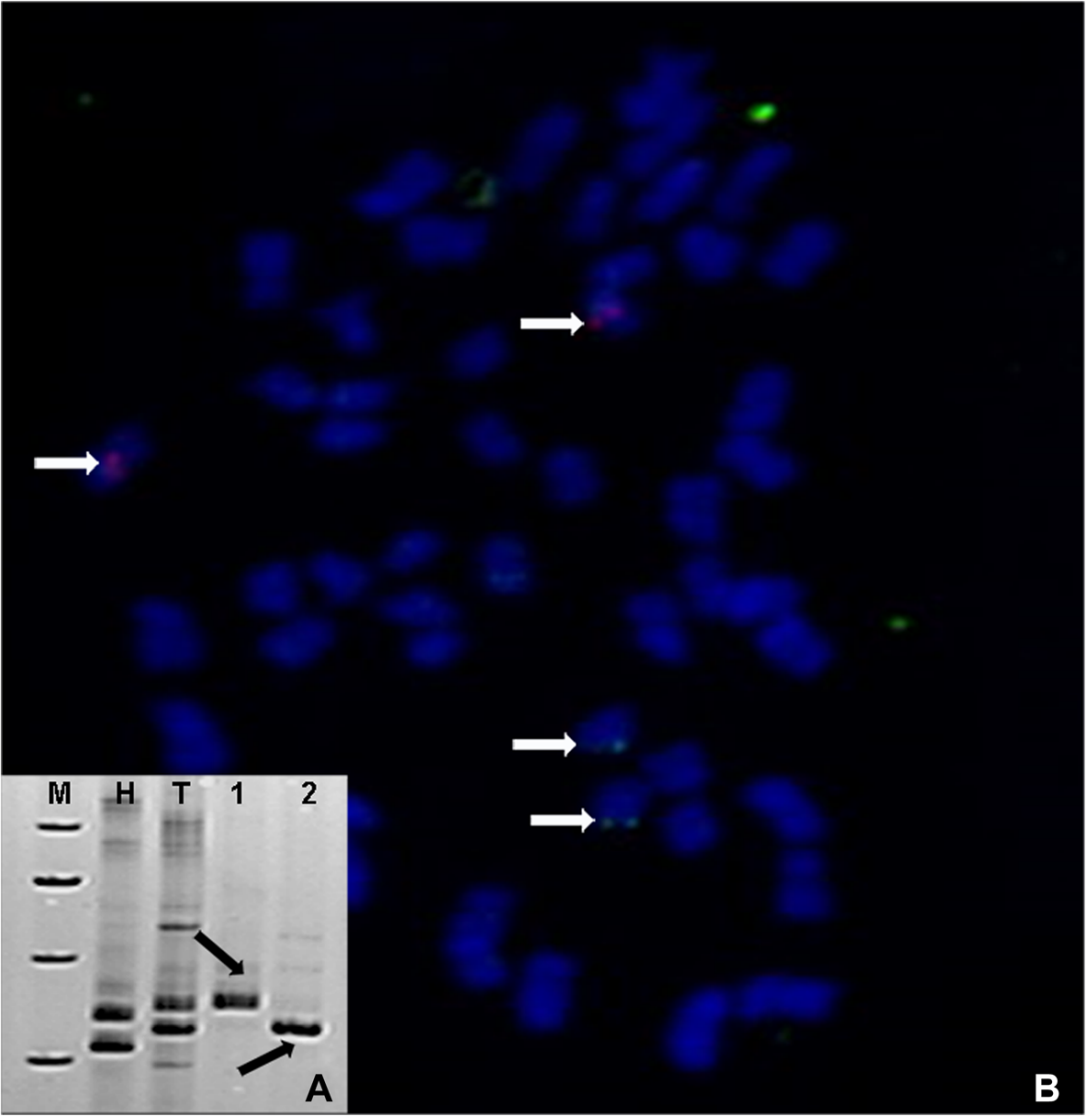
Identification of screening BAC library with NAU2251 and the loacaction of postive BAC clone with BAC-FISH. **a** Identification of the BAC clone z84A22 (lane 3) containing the polymorphic locus of NAU2251-165, and 259 L20 (lane 4) containing the polymorphic locus of NAU2251-155 between TM-1 and Hai7124 by SSR marker NAU2251. Lanes 1–4 were Hai7124, TM-1, z84A22, 259 L20 respectively. **b** FISH image showed that the signals of the polymorphic allele BAC 259 L20 (green signals, arrows) and the signals of polymorphic allele BAC z84A22 (red signals, arrows) were located on A12 and D12 homoeologous chromosomes. M: Marker, H: Hai7124, T: TM-1,1: Z84A22,2: 259 L20

**Fig. 3:**
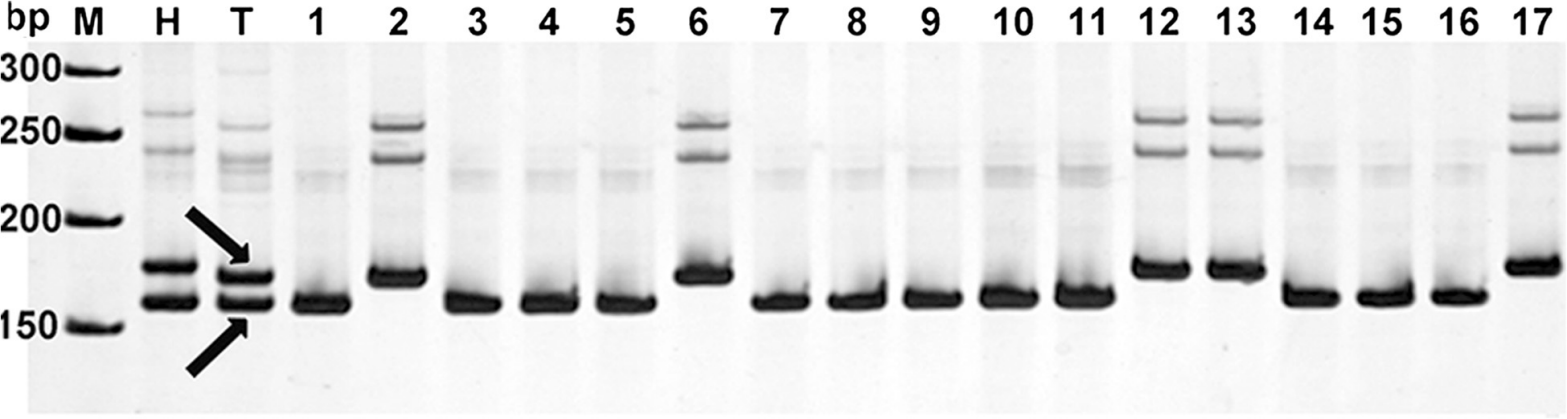
Positive BAC clones of NAU3441-180. M: Marker; H: Hai 7124; T: TM-1; 1: 19 N16; 2: 52A5; 3: 145E5; 4: 273 J12; 5: 282G8; 6: 318I15; 7: 367 M17; 8: 30P23; 9: 34G22; 10: 91 L21; 11: 363Q13; 12:367 M15; 13: 30 N23; 14: 30O23; 15: 34 J24; 16: 91B21. Individuals can be clearly defined as carrying the alleles of either parent1 (Hai7124) or parent2 (TM-1). The BAC clones that have the band NAU3441-180 the same as the parent 2 (TM-1) include 4 clones (lane2,6,12,13) and the another clones that have the band NAU3441-160 also the same as the parent2 (TM-1) include 12 clones(lane1,3-5,7-11,14-16)

A12 and D12 form a pair of homoeologous chromosomes, and previous research confirmed the presence of large homologous segments shared between them. BACs can usually be distinguished using SSR markers based on polymorphic loci. For example, the SSR marker dPL0240 produced two fragments in *G. barbadense* cv. Hai7124 and two in *G. hirsutum* acc. TM-1 (Fig. 1). One was a polymorphic locus, dPL0240_160 in Hai7124 and dPL0240_155 in TM-1, which mapped to chromosome 12. Eight BACs amplified using dPL0240 produced identical bands in TM-1. These BACs were mapped to A12, again based on the polymorphic marker. The other fragment generated by the SSR marker consisted of two polymorphic alleles, as observed with the SSR marker NAU2251 [27]. One polymorphic allele identified as NAU2251_165 in Hai7124 and NAU2251_170 in TM-1 was mapped to A12, while the other was mapped to D12 by NAU2251_155 in Hai7124 and NAU2251_160 in TM-1. Two types of BAC clones were identified by the SSR marker NAU2251. According to the polymorphic allele, BAC clone z84A22 (Fig. 2a, lane 3) contained the polymorphic locus NAU2251_165, and 259 L20 (Fig. 2a, lane 4) contained the polymorphic locus NAU2251_155 between TM-1 and Hai7124 identified using SSR marker NAU2251. BAC clones z84A22 and 259 L20 were therefore mapped to A12 and D12. Probe FISHing confirmed that BAC clones z84A22 and 259 L20 mapped to a pair of chromosomes (Fig. 2b). Additionally, the SSR marker produced two alleles in another case, one at a polymorphic locus, and the other at a monomorphic locus. The SSR marker NAU3441 produced two PCR fragments in both *G. barbadense* cv. Hai7124 and *G. hirsutum* acc. TM-1 (Fig. 3), one of which was co-dominant and polymorphic (NAU3441_180 in Hai7124 and NAU3441_170 in TM-1) and mapped to 12D [33]. The other NAU3441_160 fragment was monomorphic between Hai7124 and TM-1 and could not be mapped as it is located on its homoeologous chromosome. BAC-FISHing confirmed that one locus originated from A12 or D12 and the other locus originated from its homoeologous chromosome [15]. The corresponding BACs were therefore distinguished using the SSR markers as described above. In total, 775 BACs were identified, with 387 from chromosome A12 and 388 from chromosome D12. These BAC sets were used for fingerprinting.

### Adjustment of FPC parameters and BAC fingerprinting

**Fig. 4:**
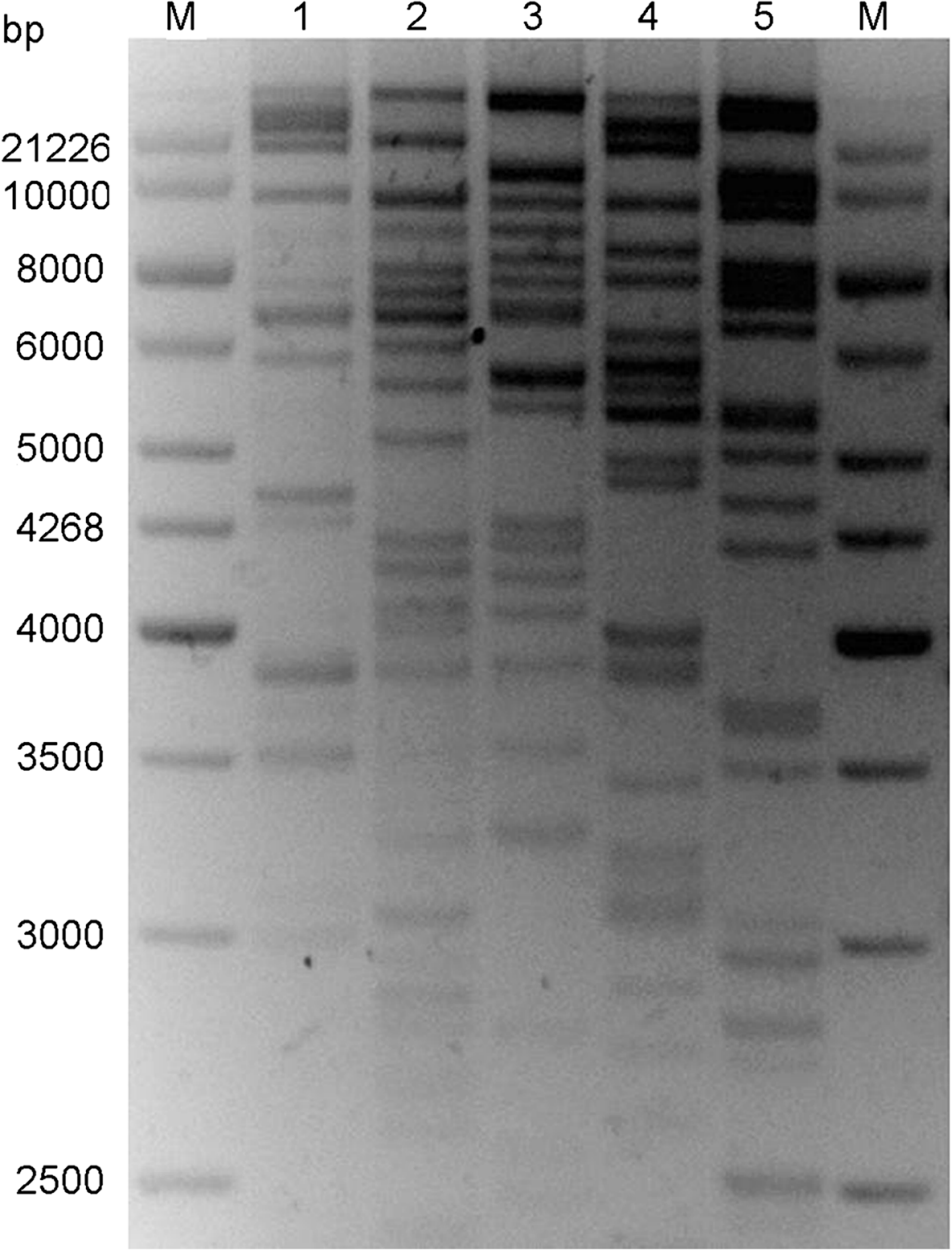
Fingerprints of five BAC clones digested with *Hin*dIII enzyme. M: Marker, 1Kb lambda ladder DNA marker + lambda DNA/*Hin*d III + *Eco*R I. 1**–**5 indicated the BAC clone. BAC DNA of five positive clones were digested with *Hin*dIII enzyme. The reaction is electrophoresed on a 4 % metaphore agarose gel and stained with ethidium bromide. The fragments of BAC clones indicate sequence specific to the BAC clone. Sizes of the marker fragments are represented in bp

BACs were digested with *Hin*dIII, and all 775 clones were fingerprinted using an agarose gel-based restriction fingerprinting method. Bands ranged in size from 1000 bp to 21,226 bp, and ranged from 10–20 pieces. A representative DNA fingerprinting gel is shown (Fig. 4). Bands were imaged using the Image3.10b software and saved as a bands file. A total of 387 clones from A12 produced 5372 fingerprint bands and 388 clones from D12 produced 4843 fingerprint bands.

**Fig. 5:**
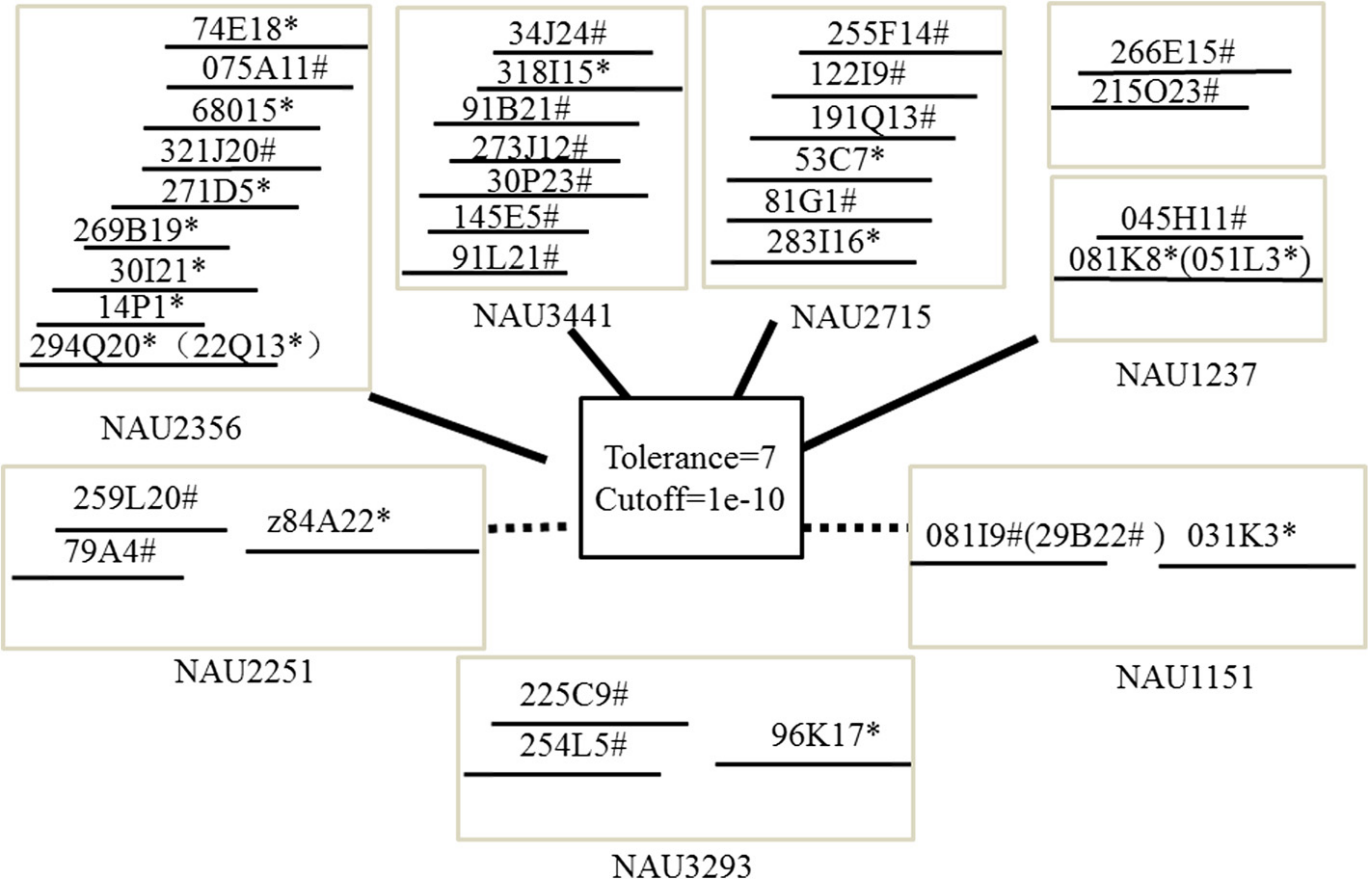
Construction of the contigs by BAC clones identified by seven SSR markers. “*”represent that the BAC clones from A12 chromosome. “#”represent that the BAC clones from D12 chromosome. Two types of contig are displayed under the parameter Tolerance = 7 and cutoff =1e-10 with solid line and dotted line. The BAC clones of seven primers can not build two contigs from A12 and D12 chromosome respectively in this parameter setting. All BAC clones built one contig that was displayed by solid line under the parameter in four primers such as NAU2356, NAU3441, NAU2715, NAU1237. All BAC clones constructed two contigs from A12 and D12 chromosome respectively that was displayed by dotted line under the parameter in three primers such as NAU2251, NAU3293, NAU1151

Tolerance and cutoff values are two important parameters in the FPC software, and it is important to set these parameters appropriately for construction of a physical map to avoid false positives. To achieve the best contig map, BACs which had seven pairs of SSR markers located on A12 and D12 were chosen for contig analysis (Table 1). Firstly, the default values of tolerance (7) and cutoff (1e-10) were tested, and all BAC clones from A12 and D12 identified as described above were combined to construct contigs. Two types of contig were generated (Fig. 5). The first type resulted from four group markers (NAU2356, NAU3441, NAU2715, NAU1237) and consisted of BAC clones from A12 and D12 (Fig. 5, solid line), which were combined to produce a single contig. The second type resulted from three group markers (NAU2251, NAU3293 and NAU1151) and consisted of BAC clones from A12 and D12 (Fig. 5, dotted line). These contigs were constructed separately for A12 and D12. It was clear that construction of contig maps for BACs from A12 and D12 separately to distinguish between the A and D subgenomes would be difficult with the default tolerance and cutoff values. We therefore adjusted these parameters in FPCV9.3 to generate a higher quality contig map for the analysis of enzyme fingerprinting. The tolerance value was set between 5 and 10, and the cutoff value was set between 1e-01 and 1e-12.

**Table 1:** BACs identified by seven pairs of SSR markers

Marker	Molecular weight	Clone ID
NAU2715(A12)	180	283I16,10G14,53C7
NAU2715(D12)	250	1 81G1, 255 F14, 79G11, 89P7
NAU3441(A12)	230	30 N23,367 M15,318I15
NAU3441(D12)	180	367 M17,91 L21,19 N16,34 J24,34G22, 273 J12, 282G8,91B21, 363Q13
NAU1151(A12)	160	194H15, 031 K3
NAU1151(D12)	170	29B22, 081I9
NAU3293(A12)	180	96 K17
NAU3293(D12)	150	225C9, 206 L15, 254 L5,192 F22
NAU2251(A12)	165	64E13, 79A4, z84A22
NAU2251(D12)	155	259 L20
NAU1237(A12)	255	051 L3, 82D24, 081 K8
NAU1237(D12)	150	215O23, **2**66E15, 45H11, 115D23
NAU2356(A12)	150	14P1, 22Q13, 69 K1, 86E19, 269B19, 271D5, 294Q20, 30I21, 68015, 084P1, 74E18, 74 F9, 96I20
NAU2356(D12)	170	075A11,321 J20,102 N2

**Fig. 6:**
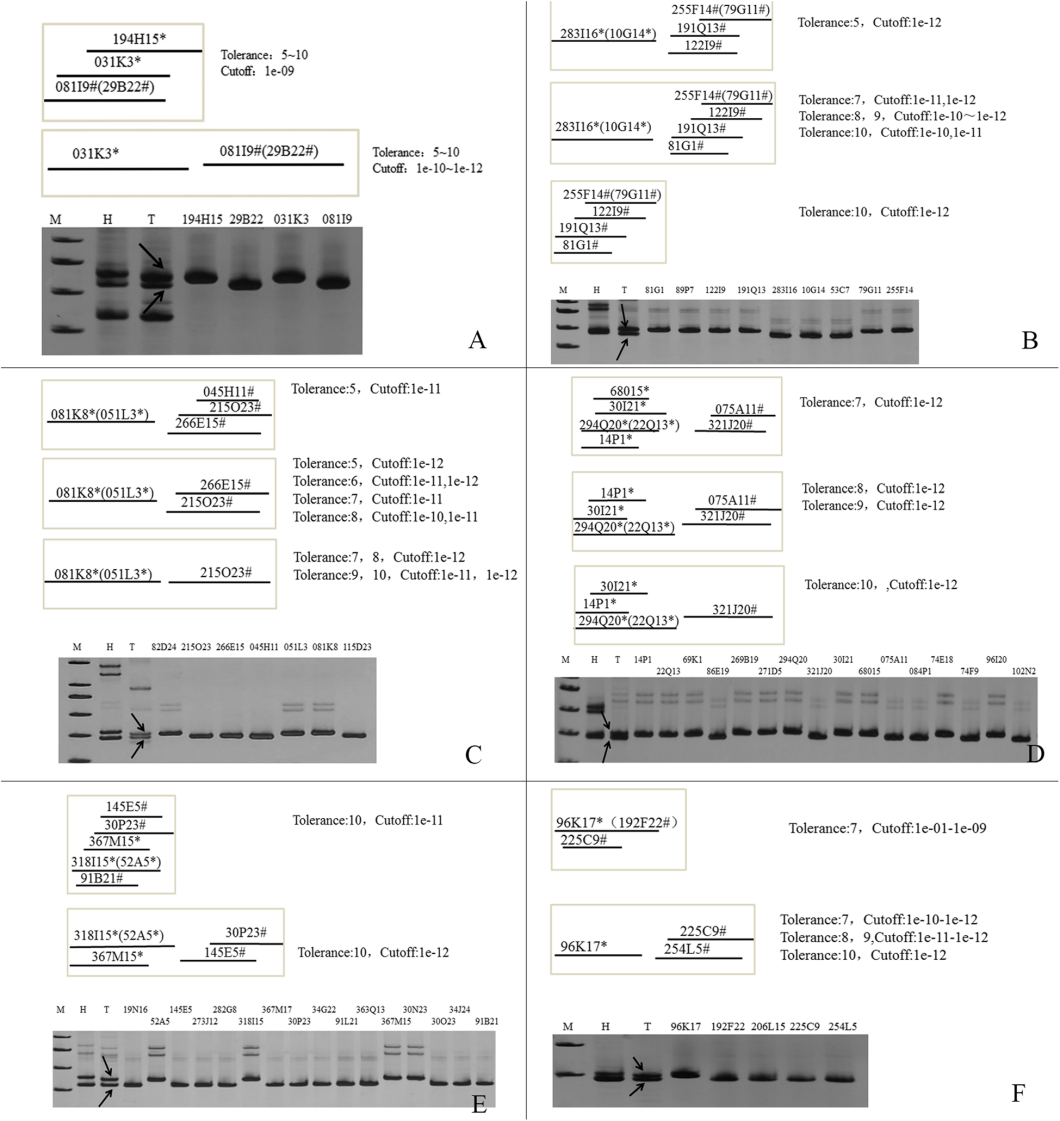
Contigs of BAC clones by six primers were built in the different parameters. Contigs were constructed by FPC software that was displayed in the top. The products of every primer were separated by PAGE that was shown in the blow (for details, see text). **a** Structure of the contigs and the amplification of BAC clones by PCR in primer NAU1151. Two types of contigs were constructed in different parameters **b** Construction of contig and PCR amplification of BAC clones in primer NAU2715. There are three types of contigs in different parameters. **c** Construction of contig and PCR amplification of BAC clones in primer NAU1237. There are three types of contigs in different parameters. **d** Construction of contig and PCR amplification of BAC clones in primer NAU2356. There are three types of contigs in different parameters. **e** Construction of contig and PCR amplification of BAC clones in primer NAU3441. There are two types of contigs in different parameters. **f** Construction of contig and PCR amplification of BAC clones in primer NAU3293. There are two types of contigs in different parameters

All BAC clones identified by NAU1151 produced two types of contigs. With a tolerance value between 5 and 10 and a cutoff value of 1e-09, the BACs from A12 and D12 combined to produce a single contig (Fig. 6a). However, when the cutoff value was increased from 1e-10 to 1e-12, BAC clones from A12 and D12 produce two contigs.

For NAU2715, with a tolerance value of 5 and a cutoff value of 1e-12, combining all BAC clones produced two contigs from A12 and D12, respectively (Fig. 6b). However when the tolerance value was increased to 7 and the cutoff value was 1e-12, BAC clones produced two contigs from A12 and D12, but BAC 81G1 appeared in D12 (Fig. 6b). Increasing the tolerance value to 10 and produced a one single contig from D12 (Fig. 6b).

Combining all BAC clones from NAU1237 produced two contigs with a tolerance value of 7 and a cutoff value of 1e-10, which could not distinguish contigs between chromosomes A12 and D12 (Fig. 6c). Two contigs for A12 and D12 were produced if the tolerance value was 5 and the cutoff value was 1e-11 (Fig. 6c). When the cutoff value was 1e-11 and the tolerance value was increased from 6 to 8, or when the tolerance value was 5 and the cutoff value was increased to 1e-12, this also produced separate contigs from A12 and D12, but BAC 045H11 disappeared from chromosome D12. When the tolerance value was 7 or 8 and the cutoff value was increased to 1e-12, the BAC clones generated separate contigs from A12 and D12, but BAC clone 266E15 disappeared from chromosome D12.

Combining all BAC clones from NAU2356 produced a single contig when the tolerance value was 7 and the cutoff value was 1e-10, which could not distinguish between A12 and D12 (Fig. 6d). With a tolerance value of 7 and a cutoff value of 1e-12, all BAC clones from NAU2356 produced two separate contigs for A12 and D12 (Fig. 6d). Increasing the tolerance value from 8 and 9 and setting the cutoff value at 1e-12 produced the contig from A12 and D12, respectively, but BAC 68015 disappeared from A12 (Fig. 6d). Increasing the tolerance value to 10 and setting the cutoff value at 1e-12 produced the contig from A12 and D12, respectively, but BAC 68015 and 075A11 disappeared from contigs (Fig. 6d).

Combining BAC clones from NAU3441 produced one contig when the parameters were set at the lower end of the range (Fig. 6e). However if the tolerance value was increased to 10 and the cutoff value was set at 1e-11, a single contig was generated (Fig. 6e), whereas with a tolerance value of 10 and a cutoff value of 1e-12, two separate contigs for A12 and D12 resulted (Fig. 6e).

Combining BAC clones from NAU3293 produced a single contig with a tolerance value of 7 and a cutoff value of 1e-01 to 1e-09 (Fig. 6f). However, increasing the cutoff value from 1e-10 to 1e-12 produced two contigs from A12 and D12, respectively (Fig. 6f). A tolerance value of 10 and a cutoff value of 1e-12 were therefore optimal.

**Fig. 7:**
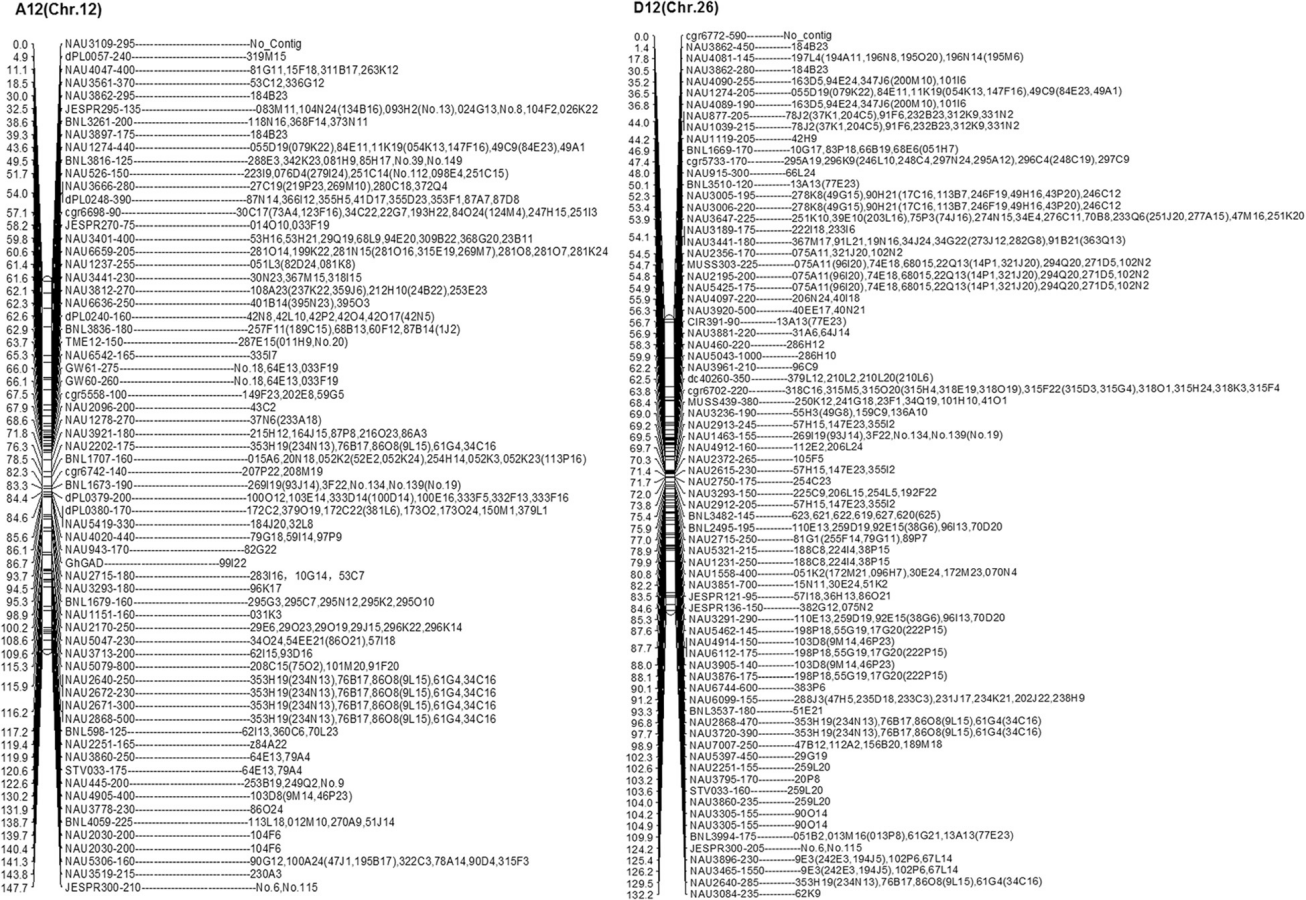
The BAC contig maps of the A12 and D12 homoeologous chromosomes. All of the BAC clones from A12 and D12 choromosomes were constructed one contig respectively. The contig of A12 chromosome was in the left and the contig of D12 chromosome in the right. The BAC clone in the contig map was displayed with the number in the library. The contig of every marker in the genetic map was correspond to the BAC clones obviously. There were no contig constructed because of absence of positive BAC clones in some markers. The contig map was clearly shown by the markers that obtained the positive BAC clones from library

Fingerprints of all 755 BACs were assembled using the optimized parameters (tolerance value 10, cutoff value 1e-12). A total of 77 contigs from A12 were generated, ranging in size from 53 kb to 155 kb, whereas 82 individual contigs were produced for D12 ranging from 61 kb to 139 kb (Fig. 7).

## Discussion

### Using BACs as a tool for constructing contig maps

Various strategies have been developed for constructing physical maps, and BAC clones are especially important for both map-based cloning and sequencing of large complex genomes containing abundant repetitive sequences and highly homologous segments. How to effectively distinguish between the homologous segments of the tetraploid cotton genome, verifying the quality of the physical map is crucial. To avoid interference from sequence similarity, BACs identified by SSR markers based on the linkage map of 12A and 12D were used for assembly in this study. Full use was made of SSR markers from high density genetic mapping to screen the BAC library and to distinguish between the A and D subgenomes. BAC clones obtained by PCR were located to A12 or D12 homoeologous chromosomes according to the published linkage map [27]. Although BAC library screening was time consuming initially, the resulting BAC contigs were accurate and this simplified the analysis later. The first integrated cytogenetic and linkage maps of homoeologous chromosomes 12A and 12D indicate that the orders of most genetic markers tested are colinear with corresponding BAC FISH signals. Although the orders and positions of polymorphic and monomorphic BACs on chromosome 12A and 12D were concordant with marker positions in the corresponding linkage map, the detailed chromosomal view of genome size variation between homoeologous chromosomes 12A and 12D was provided that show 1.3-fold size variation [35]. Furthermore, upon integrating with molecular markers, the BAC contig map of A12 and D12 will facilitate map-based cloning of QTLs or genes associated with important agronomic traits and marker-assisted selection, as well as comparative studies for analysis of the evolution of cotton genomes using sequencing of homologous segments.

### Improving contig map quality

The quality of the BAC library DNA is important for sharp, clear fingerprinting maps, and several steps in the preparation of BAC DNA are critical to the success of the process. Due to the low copy number, at least 3 ml of cell culture should be used for DNA preparation, and both cell growth and BAC production should be adequate. The cell pellet should be thoroughly suspended and cell lysis should proceed for less than 5 min. For construction of contigs, the tolerance and cutoff parameters were highly influential. We tested cutoff values from 1e-01 to 1e-12 and tolerance values from 5 to 10, which span the range used widely for agarose-based enzyme fingerprinting. A tolerance value of between 3 and 5 and a cutoff value between 1e-30 and 1e-50 are generally used in HICF fingerprint analysis [36]. A tolerance value of 4 and a cutoff value from 1e-20 to 1e-04 were selected for the BIBAC contig map, which contained at least five shared clones [37], and a tolerance value of 8 and a cutoff value of 1e-10 were used in the *Gossypium raimondii* D-genome physical map [38]. In this study, we adjusted the parameters in the FPCV9.3 software to improve the quality of the contig map for analysis by enzyme fingerprinting using homologous BACs. A tolerance value of 10 and a cutoff value of 1e-12 were found to be the optimal parameters.

## Conclusions

In this study, we successfully constructed a BAC contig map of the homoeologous chromosome 12 of *G. hirsutum* TM-1. The BAC-based method was an effective strategy for construction of the cotton physical map, but further work is necessary to improve the contig map. Even so, this work introduces a novel method for combined analysis of homoeologous chromosomes, and could provide an important framework for sequencing of the cotton genome. This work could also facilitate research into the evolution of the cotton genome through sequencing of homologous segments and comparison of genome sequences with BAC sequences. This could also generate information on the genome formation and evolutionary processes involved in cotton polyploidization.

## Methods

### Source BAC library and BAC library screening

A BAC library constructed from *G. hirsutum* cv. Texas Marker-1 (TM-1) was used in this study [27]. The library was constructed from cotton DNA partially digested with *Hin*d III and incorporated into the BAC vector pIndigBAC-5. The library consists of 147,456 clones with an average insert size of 122.8 kb that ranges from 97 to 240 kb. Approximately 96 % of the clones contain inserts over 100 kb, therefore this library represents 7.4 haploid genome equivalents in theory, based on an AD genome size of 2425 Mb. Clones were stored in 384-well plates and arrayed into multiplex pools for rapid and reliable library screening. BAC screening was carried using four-round PCR using SSR markers selected from the A12 and D12 high-density genetic maps derived from populations of the tetraploid *Gossypium* species.

### BAC-DNA isolation and fingerprinting

BAC clones were inoculated into 96-well 2.2 mL plates, and each well contained 1.5 mL of 2xYT medium (12.5 μg/mL CM). Plates were covered with sealing film and incubated at 37 °C for 20–24 h on a shaker. BAC DNA was isolated using standard alkaline lysis [39], digested with *Hin*dIII, and subjected to 1 % agarose gel electrophoresis at 40 V for 16 h. Restriction fragment identification was performed using IMAGE 3.10b software [40] with extensive manual editing. Fragments ranging from 53 to 155 bases were used for contig assembly. Bands derived from the BAC vector (pIndigBAC-5) and BACs containing less than five bands were manually deleted from the image files.

### BAC contigs assembly

The computer program FPC V9.3 (http://www.agcol.arizona.edu/software/fpc/) was used to assemble the physical map contigs from the BAC fingerprints. A series of tests were conducted in which fingerprints of a set of overlapping clones were compared using different tolerance values (from 5 to 10) and cutoff values (from 1e-01 to 1e-12). Based on these results, a tolerance of 10 and a primary cutoff of 1e-12 were selected for contig assembly.
